# The clinical significance of inflammatory mediators in predicting obesity and progression-free survival in patients with adult-onset Craniopharyngioma

**DOI:** 10.1186/s12885-024-12548-4

**Published:** 2024-07-04

**Authors:** Youchao Xiao, Wentao Wu, Fangzheng Liu, Yanfei Jia, Lu Jin, Ning Qiao, Kefan Cai, Siming Ru, Lei Cao, Songbai Gui

**Affiliations:** Department of Neurosurgery, Beijing Tiantan Hospital, Capital Medical University, Beijing, 100070 China

**Keywords:** Craniopharyngioma, Inflammatory mediators, Leptin, Obesity, Progression-free survival

## Abstract

**Background:**

Craniopharyngioma (CP) is a rare malformational tumor characterized by high rates of recurrence and morbid obesity. However, the role of inflammatory mediators in obesity and the prognosis of patients with CP remains unknown. Therefore, the present study aimed to analyze associations of inflammatory mediators with weight-related outcomes and the prognosis of patients with CP.

**Methods:**

A total of 130 consecutive patients with CP were included in this study. The expression levels of seven inflammatory mediators and the plasma leptin concentration were investigated. Clinical parameters, weight changes, new-onset obesity, and progression-free survival (PFS) were recorded. The relationships between inflammatory mediators, clinicopathologic parameters, weight-related outcomes, and PFS were explored.

**Results:**

Compared with those in normal pituitary tissue, the expressions of inflammatory mediators in tumor tissue were higher. Higher expression levels of CXCL1 and CXCL8 were identified as independent risk factors for significant weight gain, and CXCL1 and TNF were identified as independent risk factors for new-onset postoperative obesity. Poor PFS was associated with higher expression levels of CXCL1, CXCL8, IL1A, IL6, and TNF.

**Conclusion:**

The present study revealed that inflammatory mediators are associated with morbid obesity in patients with CP. Inflammatory mediators may be the critical bridge between elevated leptin and weight-related outcomes. Additionally, PFS was associated with the expression of inflammatory mediators. Further research is needed to elucidate the underlying mechanisms of inflammatory mediators and their potential as targets for novel therapies for CP.

**Supplementary Information:**

The online version contains supplementary material available at 10.1186/s12885-024-12548-4.

## Introduction

Craniopharyngioma (CP) is a rare malformational tumor mainly located in the sellar / parasellar area, accounting for 0.5–2.5 new cases per 1 million people per year globally [1]. However, despite low-grade histological malignancy (WHO grade I) and high survival rates [2], survivors often experience obesity and tumor recurrence, which significantly adversely affect their quality of life [3].

The hypothalamus acts as the balancing center for metabolic homeostasis, linking direct synaptic connections to limbic systems that mediate motivation to eat and process reward [4]. Mechanistically, neurons within hypothalamic nuclei maintain energy homeostasis by responding to nutrient signalling and hormone binding, such as leptin, insulin, ghrelin, and cholecystokinin [5]. Injury to the hypothalamus can disrupt the delicate balance between energy intake and expenditure [4]. Due to the proximity of CP to the hypothalamus and the high probability of hypothalamic damage from the tumor and treatment, hyperphagia and morbid obesity (occurring in up to 50% of cases) in CP have traditionally been attributed to physical hypothalamus damage caused by a combination of tumor invasion and treatments [1].

However, a high proportion of CP patients (more than 50%) with proven hypothalamic integrity characterized by no hypothalamic involvement (HI) and a normal floor of the third ventricle develop obesity postoperatively [6], indicating that specific unrevealed mechanisms also contribute to obesity in patients with CP, except for physical damage to the hypothalamus. Two independent studies supported the idea that injecting cyst fluid of CP into the brain of model animals contributes to the pathogenesis of obesity [7, 8]; these results help us understand the obesity of CP from the perspective of biofactors in cyst fluid secreted by the tumor. While the cyst fluid is rich in various growth factors and inflammatory mediators, including interleukin (IL)1 A, IL1B, IL6, IL10, C-X-C motif chemokine ligand (CXCL) 1, CXCL8, and tumor necrosis factor-alpha (TNF) [9, 10], and these two studies did not reveal which specific substances play a role in promoting obesity. Therefore, the present study first aimed to explore the relationship between the expression levels of these seven inflammatory mediators and weight changes, which will be helpful for subsequent experiments and drug development.

Consistent with previous research [11–13], one of our previous studies revealed that hypothalamic resistance to circulation hormones, such as leptin, contributes to obesity [14]. Patients with CP exhibit abnormally elevated preoperative leptin relative to fat mass and fail to suppress food intake or increase energy expenditure effectively, indicating the existence of leptin resistance in patients with CP [14]. Unfortunately, which factors cause hyperleptinemia and leptin resistance in patients with CP is still unknown. Emerging evidence indicates that activated glial cells secrete various cytokines and inflammatory mediators, which could impair the sensitivity of hypothalamic neurons to peripheral metabolic signals, such as leptin and insulin, ultimately resulting in metabolic dysfunction [15, 16]. However, it remains unclear whether inflammatory mediators secreted by tumor cells contribute to leptin resistance characterized by elevated plasma leptin in patients with CP, consequently leading to morbid obesity. Therefore, the present study also explored the correlation between the expression levels of inflammatory mediators and plasma leptin concentrations in CP, which could further provide evidence for supporting crass talking among inflammatory mediators, leptin, and the hypothalamus.

Although various treatment modalities, including surgery, irradiation, cyst aspiration, and intracystic therapies, have shown efficacy in improving the prognosis of patients with CP [1], reducing the rate of tumor recurrence and improving the ability to predict the prognosis of patients are still urgent problems in clinical practice. Numerous studies have evaluated the value of inflammatory mediators in predicting the prognosis of various tumors, including laryngeal squamous cell carcinoma [17], colorectal cancer [18], and gastric cancer [19]. However, studies on the associations between these seven inflammatory mediators and CP are rare. Thus, the present study also aimed to reveal the associations among these seven inflammatory mediators, clinical characteristics, and progression-free survival (PFS) in patients with CP.

## Patients and methods

### Patient selection

A single-center cohort study was conducted on patients diagnosed with CP who underwent endoscopic endonasal transsphenoidal surgery at Beijing Tiantan Hospital between January 2019 and March 2022. Patient selection was based on the following criteria: (I) age ≥ 18 years at the time of diagnosis, (II) primary CP without prior history of irradiation or cyst aspiration, (III) confirmed availability of histological diagnosis, (IV) presence of well-preserved tumor tissue for RNA testing and availability of plasma samples for enzyme-linked immunosorbent assay (ELISA) testing, and (V) minimum follow up period of 1-year. All procedures in our study followed the ethical standards of the institutional and national research committees and the Declaration of Helsinki. The institutional review board of Beijing Tiantan Hospital approved this study. Informed consent was obtained from all subjects involved in the study.

### Clinical data and definition

The baseline characteristics and demographics were obtained from the hospital medical records. Patients were followed from primary surgery until secondary surgery, administration of radiotherapy, death, or March 2023, whichever came first. Based on preoperative magnetic resonance imaging (MRI), the maximum diameters of the tumor and cyst in each of the three dimensions were defined as tumor size and cyst size, respectively. The tumor volume and cyst volume were approximately calculated using the following formula: volume = 4/3 × π × (a/2 × b/2 × c/2), where a, b, and c represent the maximum diameters of the tumor in each of the three dimensions [20]. A tumor with a cystic component comprising more than 50% of the total volume was classified as a cystic tumor [14, 20].

The extent of resection was assessed based on surgical videos and postoperative MRI and was categorized as gross total resection (GTR, 100% removal), subtotal resection (STR, ≥ 95% removal), or partial resection (PR, < 95% removal) [21]. The latter two were combined into the non-gross total resection (NTR) group in the statistical analysis. The preoperative and postoperative MRIs were used to determine the degree of preoperative and postoperative HI, respectively. The degree of HI, which included severe hypothalamus involvement (HI) (grade 2), mild HI (grade 1), and no HI (grade 0), was assessed using Puget’s grading system [22]. Based on body mass index (BMI), body weight status was categorized as obesity (BMI ≥ 30 kg/m2), overweight (BMI ≥ 25 kg/m2), or normal weight (BMI < 25 kg/m2). Consistent with our previous study, postoperative weight changes ≥ 5% were considered clinically significant [14, 20]. Hormone deficiency was assessed based on principles established in previous research [23]. PFS was defined as the time from primary surgery to recurrence or death or the last follow-up, whichever occurred first.

### Assay of inflammatory mediator expression in tumor tissue

The tumor specimens of 130 patients with CP and 11 normal pituitary tissue samples were collected during surgical procedures and quickly stored at -80℃. Total RNA was extracted from the samples using Steadypure universal RNA extraction kit (Accurate Biotechnology, Hunan, China, Cat No. AG21017). The primers used for reverse-transcription quantitative polymerase chain reaction (RT-qPCR) amplification are listed in Table 1. SYBR Green qPCR Mix (Biosharp Biotechnology, BL698A) was used to perform qPCR on QuantStudio 5 RealTime PCR System following the manufacturer’s protocol. The housekeeping gene GAPDH was used for normalization. The relative mRNA expression was determined using the 2^−ΔΔCT^ method and was expressed as log2 (X + 1). Each sample was tested in triplicate.

**Table 1:**
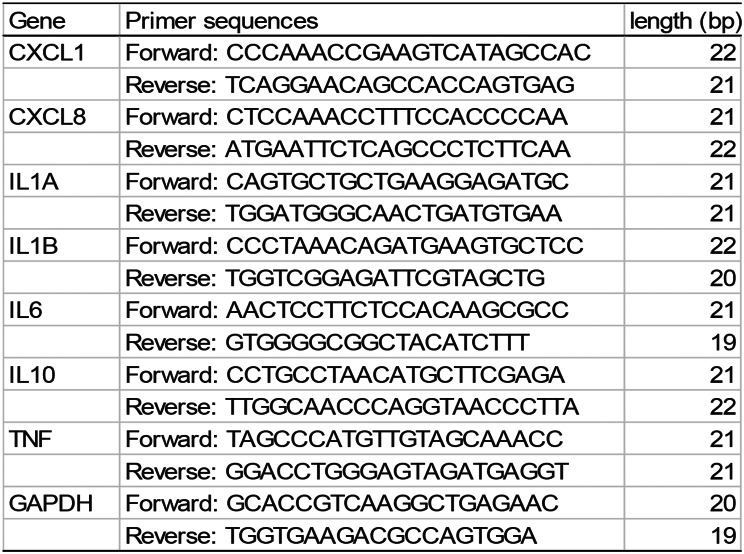
Gene-specific primers used in the RT-qPCR

### Measurement of plasma leptin concentration

Peripheral blood samples were obtained from patients with CP before tumor surgery. The samples were centrifuged at 3000 rpm for 2 min at 4 °C, and the plasma was collected and stored at -80 ℃ until the ELISA test. The plasma leptin concentration was measured using the ELISA method according to the manufacturer’s protocol (Peprotech, Inc., Cat No. 900-K90).

### Statistical methods

Patients were categorized into high- and low-expression groups according to the median levels of inflammatory mediators. Univariate and multivariate logistic regression analyses were performed to calculate the unadjusted odds ratio (uOR) and the adjusted odds ratio (aOR); And clinical parameters, including age, sex, histopathologic subtype, calcification, preoperative HI, postoperative HI, the extent of surgical resection, and preoperative body mass index, were adjusted for in the multivariate logistic analysis. The Pearson correlation test was also used to estimate the correlation between inflammatory mediator expression and leptin concentration. Parametric variables were analyzed using Student’s t-test, while nonparametric variables were analyzed using the Mann-Whitney U-test to compare between groups. Categorical variables were compared using Chi-square and Fisher’s exact tests. The Spearman correlation test performed correlation analysis between gene expression, size, and volume (both tumor and cyst). The Kaplan-Meier (K–M) curves and log-rank tests were employed to validate the survival rates between the low- and high-expression groups. A *P*-value < 0.05 was considered to indicate statistical significance. Furthermore, all the statistical analyses were performed, and the results were visualized using SPSS 24 (SSPS, Inc., Chicago, USA) and GraphPad Prism 9 (GraphPad Software, Inc., La Jolla, CA, USA).

## Results

### Patient characteristics

**Fig. 1:**
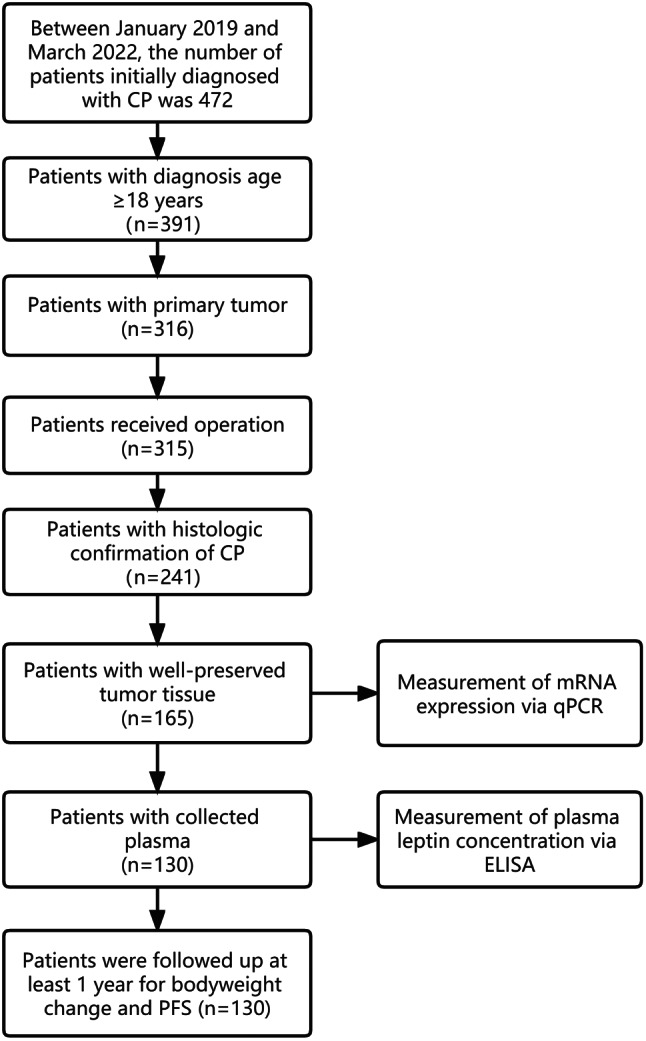
The flowchart of patient selection

Figure 1 visually illustrates the patient selection process, while Table 2 summarizes patient characteristics. This study included 130 participants (71 males and 59 females) with a mean age of 43.92 ± 12.52 years. Histological analysis confirmed 98 cases of adamantinomatous craniopharyngioma (ACP) and 32 cases of papillary craniopharyngioma (PCP). The mean duration of follow-up was 22.67 ± 9.29 months. Calcification was observed in 61.5% of the cohort (*n* = 80). Preoperative hydrocephalus and preoperative visual field damage were present in 26.2% (*n* = 34) and 17.7% (*n* = 23) of patients, respectively. Regarding preoperative body weight, 60 patients had normal weight, 56 were overweight, and 14 were obese. Gonadotropic hormone deficiency (*n* = 34, 26.2%) and antidiuretic hormone deficiency (*n* = 32, 24.6%) were the two most common preoperative hormone deficiencies in patients with CP. The incidence of other hormone deficiencies, including growth hormone deficiency (*n* = 19, 14.6%), thyroid-stimulating hormone deficiency (*n* = 16, 12.3%), and adrenocorticotropic hormone deficiency (*n* = 12, 9.2%), was less than 15%.

**Table 2:**
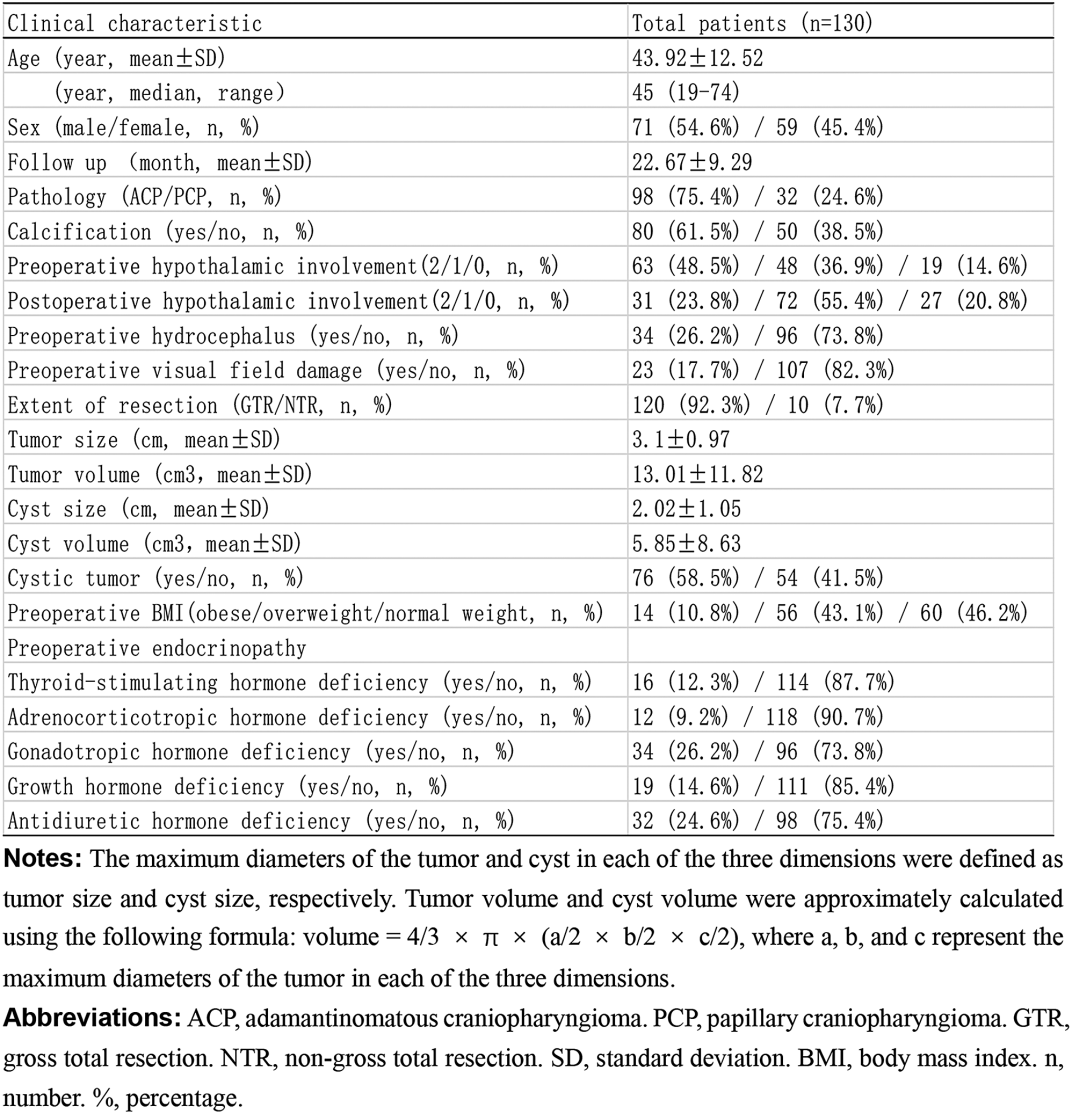
Clinical characteristics of patients with craniopharyngioma

On preoperative MRI, severe HI (grade 2) was observed in approximately half of the patients (*n* = 63, 48.5%), while mild HI (grade 1) and no HI (grade 0) were observed in 36.9% (*n* = 48), and 14.6% (*n* = 19) of patients, respectively. Based on postoperative MRI, severe HI, mild HI, and no HI were postoperatively observed in 31 patients, 72 patients, and 27 patients, respectively. The mean tumor size was 3.1 ± 0.97 cm, and the mean tumor volume was 13.01 ± 11.82 cm3. Moreover, the mean size and mean volume of the cysts were 2.02 ± 1.05 cm and 5.86 ± 8.62 cm3, respectively. Among the 130 cases, 76 tumors were identified as cystic tumors. Finally, GTR was achieved in 120 patients (92.3%), and NTR was achieved in 10 patients (7.7%).

### Association between inflammatory mediators, weight gain, new-onset obesity, and leptin concentration

**Fig. 2:**
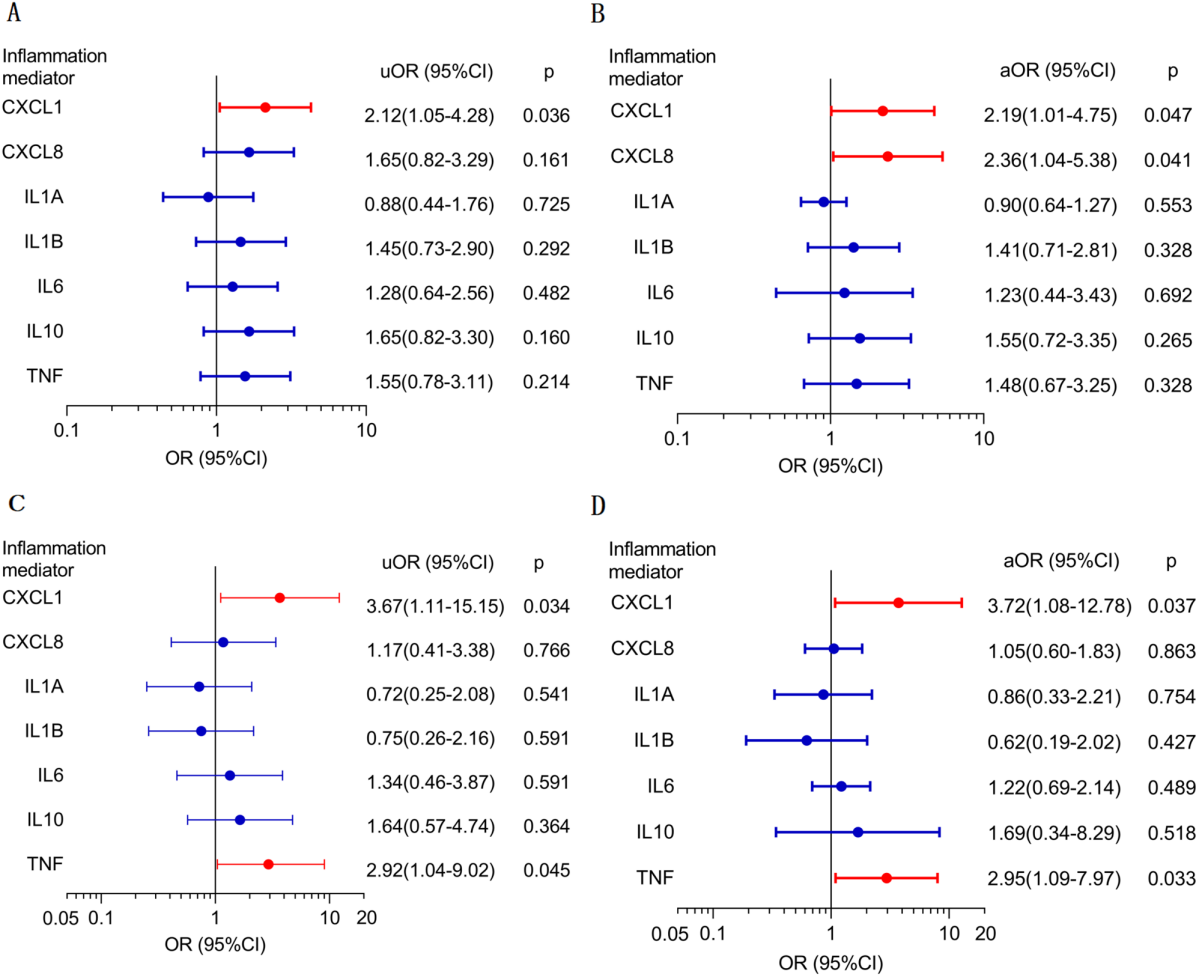
Univariate logistic analysis for (**a**) significant weight gain and (**c**) new-onset obesity. Multivariate logistic analysis for (**b**) significant weight gain and (**d**) new-onset obesity. Clinical parameters, including age, sex, histopathologic subtype, calcification, preoperative hypothalamic involvement, postoperative hypothalamic involvement, the extent of surgical resection, and preoperative body mass index, were adjusted for in the multivariate logistic analysis. uOR, unadjusted odds ratio. aOR, adjusted odds ratio

According to the univariate logistic regression analysis, significant weight gain (weight change ≥ 5%) was associated with higher expression of CXCL1 (Fig. 2A; unadjusted odds ratio (uOR) = 2.12; 95% confidence interval (CI), 1.05–4.28; *P* = 0.036). New-onset postoperative obesity was associated with high expressions of CXCL1 (Fig. 2C; uOR = 3.67; 95% CI, 1.11–12.15; *P* = 0.034) and TNF (Fig. 2C; uOR = 2.92; 95% CI, 1.04–9.02; *P* = 0.045). Multivariate logistic regression analysis adjusted for age, gender, preoperative HI, postoperative HI, and preoperative body mass index (BMI) was conducted to explore the independent relationships among inflammatory mediators, significant weight gain, and new-onset obesity. The results revealed that high expressions of CXCL1 (Fig. 2B; adjusted odds ratio (aOR) = 2.19; 95% CI, 1.01–4.75; *P* = 0.047) and CXCL8 (Fig. 2B; aOR = 2.36; 95% CI, 1.04–5.38; *P* = 0.041) were independent risk factors for significant weight gain, while high expression levels of CXCL1 (Fig. 2D; aOR = 3.72; 95% CI, 1.08–12.78; *P* = 0.037) and TNF (Fig. 2D; aOR = 2.95; 95% CI, 1.09–7.97; *P* = 0.033) were found to be independent risk factors for new-onset postoperative obesity.

**Fig. 3:**
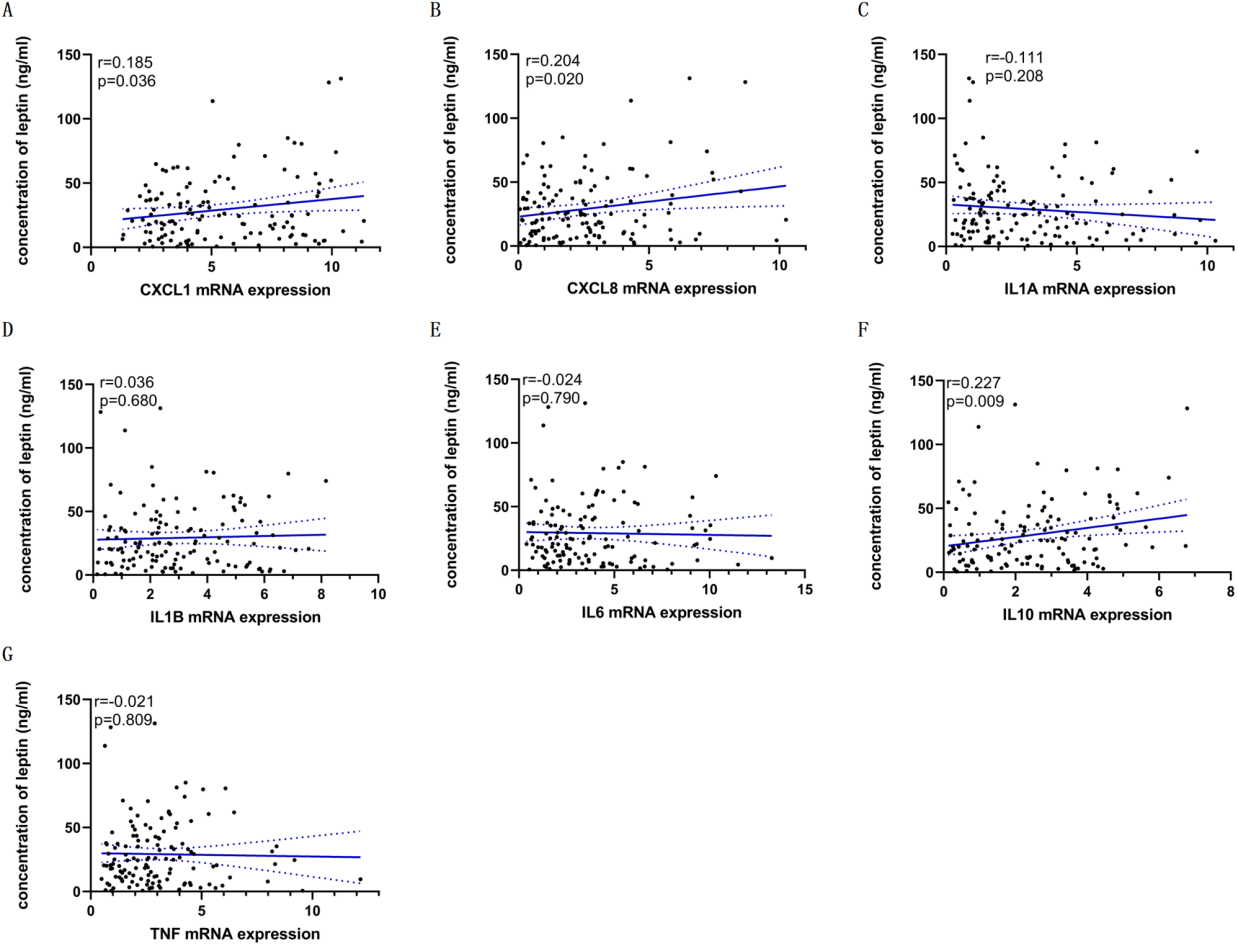
Correlations between inflammatory mediators expression and plasma leptin concentration

Leptin, a cytokine secreted by fat cells that regulates energy balance, is abnormally elevated relative to the degree of obesity in patients with CP [14]. However, the underlying mechanisms of leptin elevation and resistance in CP remain uncertain. Therefore, we attempted to explain this abnormal elevation by investigating the relationship between the inflammatory mediators and plasma leptin. The mean plasma leptin concentration was 29.18 ± 25.56 ng/ml, ranging from 0.5 ng/ml to 131.3 ng/ml. The plasma leptin concentration was positively correlated with expressions of CXCL1 (Fig. 3A, *r* = 0.185, *P* = 0.036), CXCL8 (Fig. 3B, *r* = 0.204, *P* = 0.020), and IL10 (Fig. 3F, *r* = 0.227, *P* = 0.009), while it did not show a significant correlation with the expression levels of IL1A (*P* = 0.208), IL1B (*P* = 0.680), IL6 (*P* = 0.790), and TNF (*P* = 0.809).

### Inflammatory mediators associate with clinical characteristics and progression-free survival

**Fig. 4:**
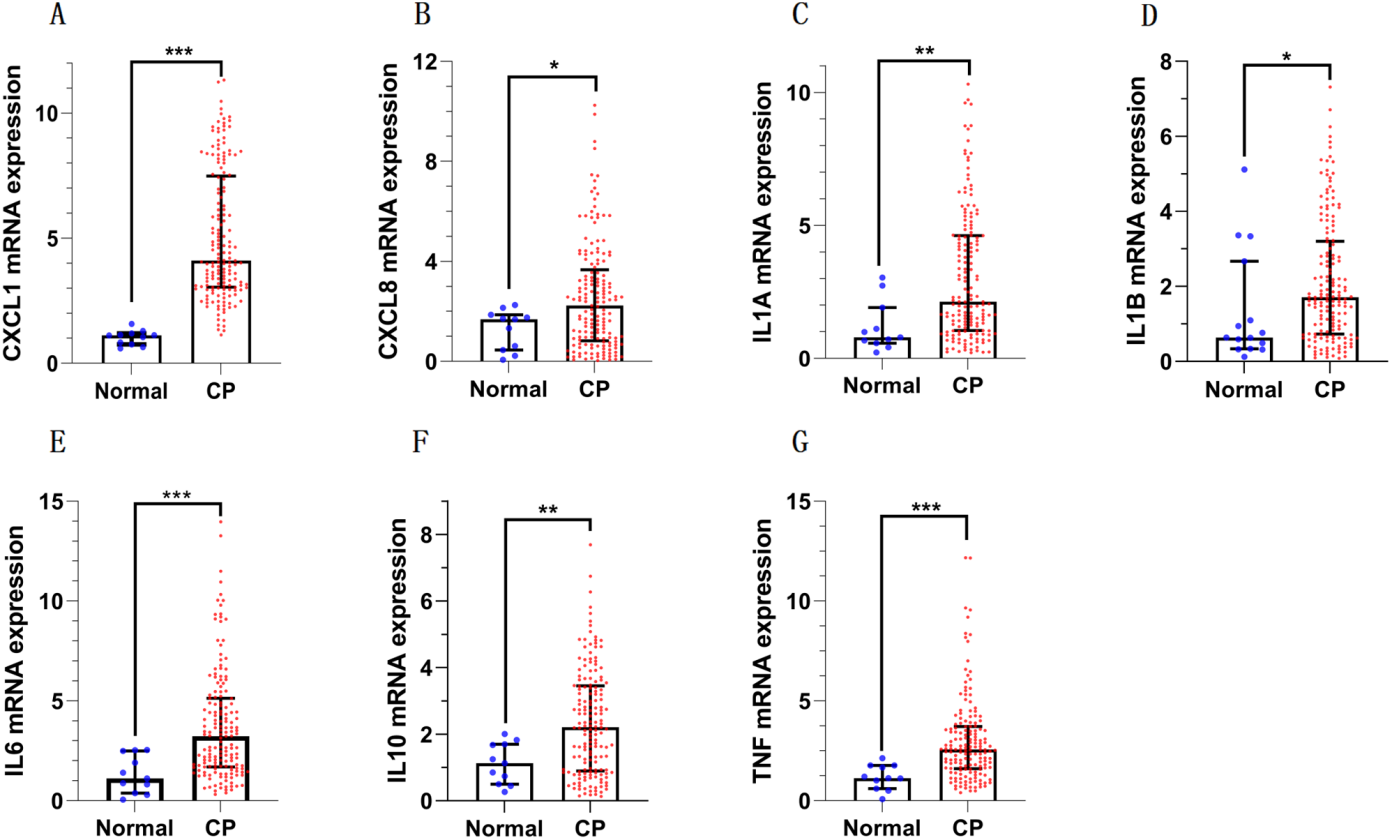
The expressions of inflammation mediators were elevated in the CP tumor tissue compared to normal brain tissue. * means *p* < 0.05, ** means *p* < 0.01, *** means *p* < 0.001

The mRNA expression levels of these seven inflammatory mediators were significantly higher in CP tumor tissue than in normal pituitary tissue (Fig. 4, and all *P* < 0.05, Mann Whitney U-test).

Table 3 depicts the associations between inflammatory meditator expression and clinicopathologic parameters. Compared with the PCP, the ACP exhibited lower levels of CXCL1, CXCL8, IL1A, and IL1B (all *P* < 0.05) but higher levels of IL10 (*P* = 0.027). Tumors with calcification exhibited lower CXCL1, CXCL8, IL1A, and IL1B (all *P* < 0.01) than those without calcification. Furthermore, severe preoperative HI was associated with higher levels of CXCL1 (*P* = 0.013) and CXCL8 (*P* = 0.043), and severe postoperative HI was also associated with higher levels of CXCL1 (*P* = 0.028). Moreover, higher IL6 expression and lower TNF expression were associated with preoperative hydrocephalus. Cystic tumors exhibited higher expression of IL1B, IL6, IL10, and TNF (all *P* < 0.05) than non-cystic tumors. Furthermore, no significant associations were detected between inflammatory meditators expression and other characteristics, including age, sex, preoperative visual field damage, and preoperative obesity. Regarding pituitary deficiency, thyroid-stimulating hormone deficiency was associated with a higher expression level of CXCL8 (*P* = 0.049), antidiuretic hormone deficiency was associated with a higher level of IL6 (*P* = 0.021), and more details were shown in Supplementary Table 1.

**Table 3:**
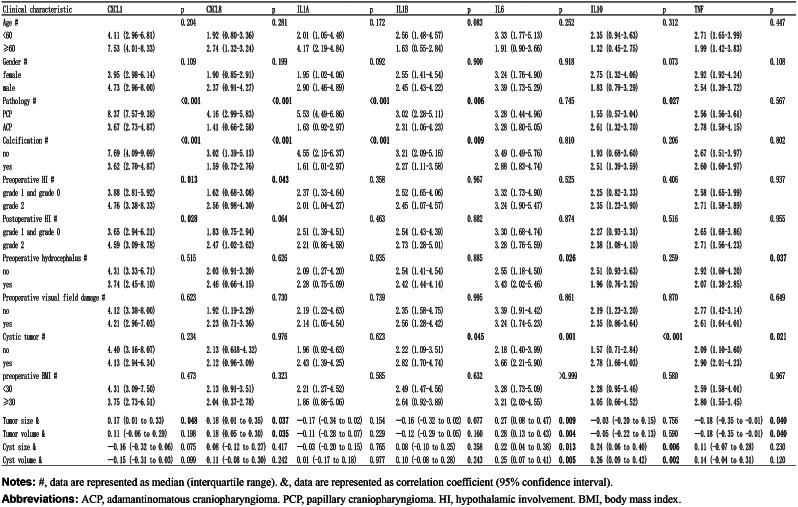
Association between clinicopathologic parameters and expression level of inflammatory mediator

As shown in Table 3, tumor size was positively correlated with levels of CXCL1 (*r* = 0.17, *P* = 0.048), CXCL8 (*r* = 0.18, *P* = 0.037), and IL6 (*r* = 0.27, *P* = 0.009). Tumor volume was positively correlated with levels of CXCL8 (*r* = 0.18, *P* = 0.037) and IL6 (*r* = 0.28, *P* = 0.004). In contrast, TNF expression negatively correlated with tumor size (*r*= -0.18, *P* = 0.040) and tumor volume (*r*= -0.18, *P* = 0.040). In terms of cyst size and cyst volume, IL6 expression was positively correlated with cyst size (*r* = 0.22, *P* = 0.013) and cyst volume (*r* = 0.25, *P* = 0.005), and IL10 expression was also positively correlated with the cyst size (*r* = 0.24, *P* = 0.006) and cyst volume (*r* = 0.26, *P* = 0.002).

**Fig. 5:**
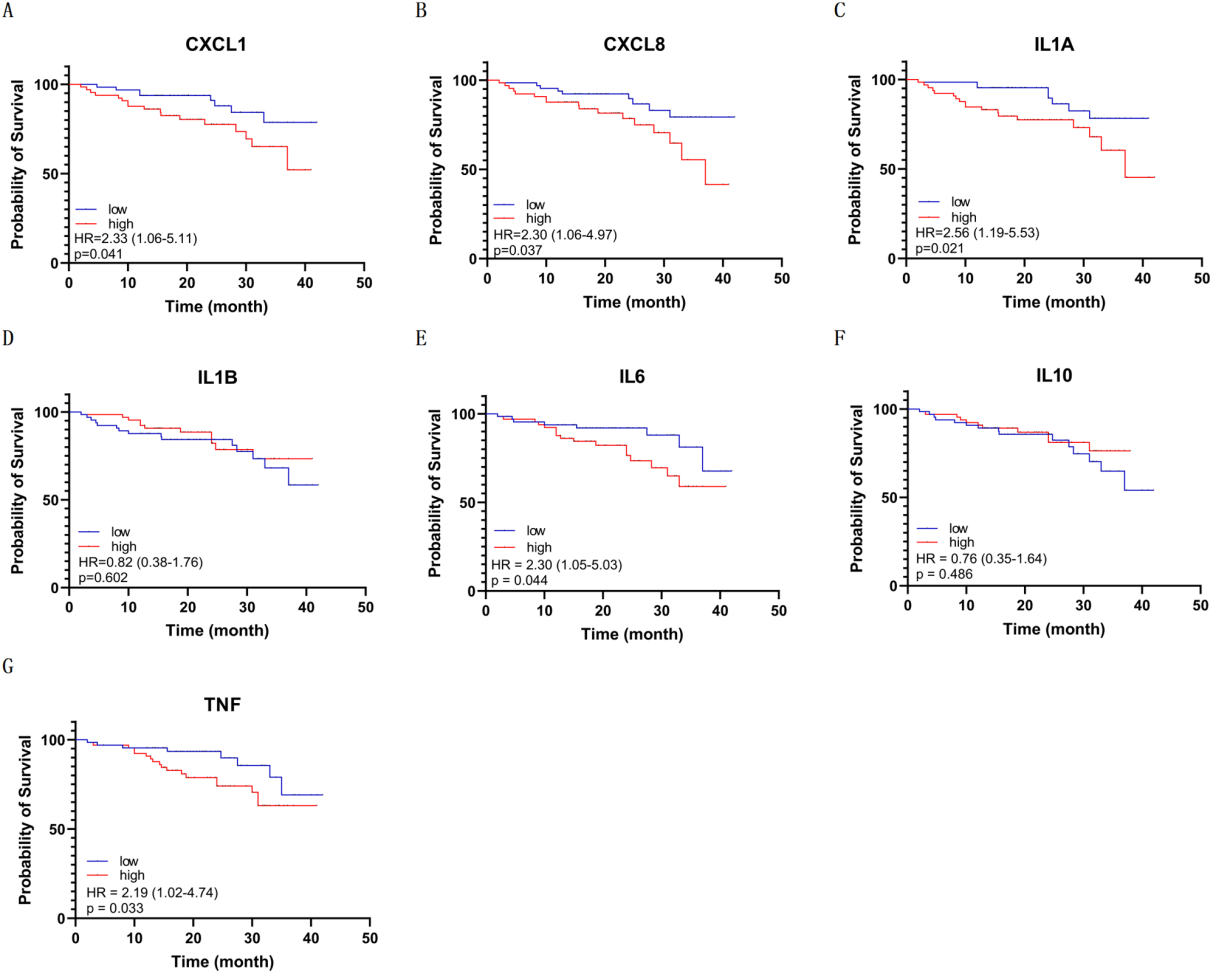
The prognostic value of inflammatory mediators expression in CP. The Kaplan–Meier curves indicate that poor PFS was associated with higher CXCL1, CXCL8, IL1A, IL6, and TNF expression. HR, hazard ratio

To determine the prognostic value of inflammatory mediators in patients with CP, we dichotomized the cohort into two groups (high and low expression groups) based on the median expression of each gene, and the details are shown in Fig. 5. The K–M curves revealed that poor PFS was associated with high CXCL1 (hazard ratio (HR) = 2.33; 95% CI, 1.06–5.11; *P* = 0.041), CXCL8 (HR = 2.30, 95% CI, 1.06–4.97; *P* = 0.037), IL1A (HR = 2.56; 95% CI, 1.19–5.53; *P* = 0.021), IL6 (HR = 2.30; 95% CI, 1.05–5.03; *P* = 0.044), and TNF (HR = 2.19; 95% CI, 1.02–4.74; *P* = 0.033) expression. However, no significant association was observed between poor PFS and the expression of IL1B (*P* = 0.602) or IL10 (*P* = 0.486).

## Discussion

Craniopharyngioma (CP), classified as a histologically nonmalignant intracerebral tumor (WHO grade I), typically exhibits favourable 5- and 10-year survival rates [2, 24]. However, the management of CP presents significant challenges due to its high recurrence rate and long-term morbidities, such as morbid obesity [25]. Recent studies have established that inflammatory mediators are tightly associated with tumor recurrence and obesity [26, 27], while these associations have not been thoroughly explored in CP. Therefore, we examined the expression profiles of inflammatory mediators in CP and investigated the potential associations between inflammatory mediators and clinical outcomes to identify targets for rational therapy.

### Inflammatory mediators, leptin, and weight-related outcomes

In recent decades, the high incidence of obesity (up to 50%) in patients with CP was widely accepted to be a consequence of physical hypothalamic damage caused by the combination of tumor invasion and treatment [28–30]. Two independent studies revealing cyst fluid contributes to obesity turned us to the obesogenic effect of inflammatory mediators riched in cyst fluid [7, 8]. The present study is the first to explore the associations between expression levels of inflammatory mediators and weight-related outcomes, including significant weight gain and new-onset obesity, in patients with CP. The result showed that higher expression levels of CXCL1 (aOR = 2.19, *P* = 0.047) and CXCL8 (aOR = 2.36, *P* = 0.041) were independently associated with significant weight gain, and higher expression levels of CXCL1 (aOR = 3.72, *P* = 0.037) and TNF (aOR = 2.95, *P* = 0.033) were independently associated with new-onset obesity postoperatively. Leakage of these inflammatory mediators causes severe chemical meningitis [31] and can trigger inflammatory activation of microglia to damage the hypothalamic neurons by inducing the production of β-amyloid [8]. In addition, cyst fluid contributes to tumor cells’ lipid metabolism disorder, which is closely associated with inflammation of the hypothalamus [31, 32]. Further evidence supports that hypothalamic inflammation can induce obesity in model animals fed a high-fat diet [33].

The hypothalamus acts as the balancing center for metabolic homeostasis and regulates energy balance through the leptin-melanocortin pathway [34]. For example, leptin binds to the receptors of hypothalamic neurons to increase metabolism and decrease food intake [35]. Physiological dysfunctions of the hypothalamus, such as insensitivity to leptin, could lead to energy imbalance [4, 13, 36–39]. Hypothalamic inflammation has been implicated in the development of obesity [15, 27], and this inflammation results in the activation of the NF-κB pathway, overexpression of suppressor of cytokine signalling 3 (SOCS3, a potential mediator of central leptin resistance), and the subsequent development of leptin resistance [35, 40]. In the present study, we found a positive correlation between leptin concentration and levels of CXCL1 (*r* = 0.185, *P* = 0.036), CXCL8 (*r* = 0.204, *P* = 0.020), and IL10 (*r* = 0.227, *P* = 0.009). These results provide preliminary data accounting for the role of inflammatory mediators in leptin elevation and obesity, and we hypothesize that CP tumor cells may induce hypothalamic leptin resistance and subsequent obesity via expressing high inflammatory mediators. Further research focusing on revealing the underlying mechanism of leptin resistance induced by inflammatory mediators in CP will facilitate the design of drugs to block the pathways associated with impaired metabolism and ameliorate obesity.

### Inflammatory mediators, tumorigenesis, and pathogenesis of cysts

Differences, including age distribution, incidence rate, pathological behavior, and, most notably, the context of tumorigenesis, exist between the two distinct tumor types (PCP and ACP) [41]. The senescence-associated secretory phenotype (SASP) means specific cell clusters undergo senescence and secrete growth factors and cytokines, and the SASP has been observed in ACPs and is associated with tumor cell growth [1]. Although the SASP phenomenon has not been observed or well characterized in PCP, the BRAF-v600e (somatic BRAF-v600e mutations primarily drive PCP) is regarded as a senescence inducer [42]. Previous studies have demonstrated that cyst fluid and solid components of CP exhibit high levels of inflammatory mediators [10]. Consistent with these findings, our study also revealed significantly higher expression of inflammatory mediators in CP than in normal pituitary tissue (Fig. 2, all *P* < 0.05). Furthermore, we reported that PCP exhibited higher expression of several inflammatory mediators, including CXCL1, CXCL8, IL1A, IL1B, and IL10, compared to ACP (Table 3). In a previous study, the IL6 high expression group with gastric cancer exhibited tumors with a higher diameter [19]; our results also indicated a positive correlation of tumor size and volume with the expression levels of CXCL1, CXCL8, and IL6, while a negative correlation was observed with TNF expression (Table 3). Therefore, these findings indicate that inflammatory mediators associated with clinical parameters play an essential role in tumorigenesis in both ACP and PCP, even in the absence of the SASP phenomenon.

The primary treatment methods for CP currently involve GTR or STR combined with radiotherapy. Nevertheless, radical surgery can lead to treatment-related damage due to the invasiveness of CP and its proximity to adjacent neural tissue, such as the hypothalamus, pituitary stalk, and optic chiasma. Reducing the mass effect of cyst fluid or solid components has become an option for temporary tumor control and delaying vision deterioration with the expectation of achieving further GTR [43]. Therefore, gaining a better understanding of cyst pathogenesis and developing more effective treatments to limit the volume of cysts are crucial. Carbonic anhydrase IX, an enzyme that causes fluid production, was revealed to be associated with the formation and size of cysts in CP [44], but the specific regulatory mechanisms of this enzyme have yet to be fully elucidated. One study demonstrated the essential role of the inflammatory response in generating cysts with highly elevated levels of inflammatory mediators [45]. The efficacy of intracystic IFN-alpha treatment has been shown to shrink cysts by reducing inflammation-mediated responses, further supporting the causal role of inflammatory mediators in cyst pathogenesis [46]. However, previous studies exploring the correlation between expression levels of inflammatory mediators, cyst size, and cyst volume have been limited by the small sample sizes (ranging from 5 to 15). Hence, the present study enrolled 130 patients (the largest known case series) and verified the higher expression of inflammatory mediators in cystic tumors and their positive correlation with cyst size and cyst volume (Table 3). These results provide further evidence supporting the potential application of anti-inflammation therapies for controlling cyst volume.

### Predictive factors of prognosis in patients with CP

Previous analyses have investigated the associations between the prognosis of CP and clinical parameters, including the number of surgical interventions, histological type, degree of HI and obesity, neuroendocrine deficiencies [1], surgical approach [47], and calcification [48]. Although these clinical factors related to prognosis provide excellent value for guiding treatment decisions, they do not fully elucidate the etiology and pathogenesis of patients with CP, thereby limiting the development of precise biomarker-based therapies.

In the era of personalized medicine, biomarkers hold promise for predicting recurrence and guiding targeted therapies in patients with CP. Therefore, numerous studies have attempted to identify biomarkers to improve prognosis prediction and develop novel targeted therapies. We previously identified integrin α6 as a biomarker associated with adverse overall survival of CP [49]. Furthermore, a study conducted by Zou et al. [50]. identified 10 hub genes for potential application in early diagnosis and therapy for ACP. Several studies have also identified the associations between the prognosis of CP and many biomarkers, including B7-H3 [51], β-catenin [52], and growth hormone receptors [53]. Moreover, another study identified four inflammatory mediators (CXCL6, CXCL10, CXCL11, and CXCL13) as hub genes with great value for targeted therapy in CP [54]. Our results revealed that high expression levels of inflammatory mediators, including CXCL1, CXCL8, IL1A, IL6, and TNF, were associated with shorter PFS in patients with CP (Fig. 5), further validating the potential value of anti-inflammation therapies in CP. Identifying prognostic biomarkers in patients with CP enables the identification of patients at high risk of recurrence and provides insights into targeted therapies based on these biomarkers.

## Conclusion

Our study demonstrated elevated expression levels of inflammatory mediators in CP, with a particularly prominent presence in the PCP. The associations of inflammatory mediators with clinical parameters indicate their potential role in the pathogenesis of tumors and cysts. In addition, inflammatory mediators were independently associated with significant weight gain and new-onset postoperative obesity. Moreover, the expression of inflammatory mediators exhibited significant prognostic value in patients with CP. Therefore, investigating therapeutic strategies targeting these inflammatory mediators holds promise for improving the prognosis of patients with CP and reducing complications, particularly leptin resistance and subsequent morbid obesity. Further studies are warranted to explore the potential of targeting inflammatory mediators as a means to optimize CP management and enhance patient outcomes.

### Electronic supplementary material

Below is the link to the electronic supplementary material.

Supplementary Material 1

## Data Availability

No datasets were generated or analysed during the current study.
