# Supplementary material for: The clinical significance of inflammatory mediators in predicting obesity and progression-free survival in patients with adult-onset Craniopharyngioma
Source: BMC Cancer. 2024 Jul 4;24:799. doi: 10.1186/s12885-024-12548-4 (PMC11229012; doi:10.1186/s12885-024-12548-4)
Supplement: Supplementary file 1 — Supplementary Material 1 [file 12885_2024_12548_MOESM1_ESM.docx]

**Supplementary Table 1:** Association between pituitary deficiency and expression level of inflammatory mediator. Data are represented as median (interquartile range).
